# Hematological and inflammatory differences associated with pipe tobacco smoking: A cross-sectional study

**DOI:** 10.1016/j.toxrep.2026.102367

**Published:** 2026-10-31

**Authors:** Osama Al-Saffar, Hamid AL-Tameemi

**Affiliations:** a Department of Medical Lab Technical, Balad Technical Institution, Middle Technical University, Baghdad, Iraq; b College of Medical and Health Techniques, University of Bilad Alrafidain, Diyala, Iraq

**Keywords:** Pipe tobacco smoking, Cytokines, Interleukin-6, Interleukin-10, Immune dysregulation, Cross-sectional study

## Abstract

Introduction: Although the systemic effects of cigarette smoking are well characterized, biological evidence on conventional pipe tobacco smoking remains limited. This cross-sectional study assessed hematological parameters and circulating cytokine profiles in exclusive pipe smokers compared with healthy non-smokers. Methods: This cross-sectional comparative study, conducted from October 2024 to July 2025, included 79 adults: 39 daily exclusive pipe smokers (21 males, 18 females; mean age 38.05 ± 14.32 years) and 40 healthy non-smokers (21 males, 19 females; mean age 38.56 ± 11.22 years). Red blood cell (RBC) and white blood cell (WBC counts were measured using an automated hematology analyzer (Sysmex XN-1000), and serum IL-6, IL-8, IL-1β, and IL-10 levels were measured by enzyme-linked immunosorbent assay (ELISA). Results: Pipe smokers had significantly higher mean RBC counts than non-smokers (5.57 ± 0.99 vs. 4.75 ± 0.79 × 10⁶/μL; mean difference: 0.82 × 10⁶/μL, 95% CI: 0.42–1.22; p < 0.0001), whereas WBC counts did not differ significantly (7.45 ± 1.84 vs. 7.16 ± 1.42 × 10 ³/μL; p = 0.443). All four cytokines were significantly higher in pipe smokers: IL-6 (99.56 ± 40.64 vs. 16.07 ± 11.41 pg/mL), IL-8 (79.29 ± 30.47 vs. 15.97 ± 9.40 pg/mL), IL-1β (274.7 ± 124.5 vs. 39.50 ± 32.97 pg/mL), and IL-10 (197.9 ± 90.10 vs. 40.33 ± 26.90 pg/mL; p < 0.0001 for all comparisons). Age was weakly associated with IL-10 (r = 0.224, p = 0.047) but not with pro-inflammatory cytokines. Conclusions: Conventional pipe tobacco smoking was associated with higher RBC counts and markedly elevated pro- and anti-inflammatory cytokine levels. These findings suggest immune and hematopoietic stimulation; however, longitudinal studies with comprehensive hematological assessments and clinical endpoints are needed to establish causality and pathophysiological significance.

## Highlights

• Pipe tobacco smoking a significant increase in red blood cell (RBC) counts (p<0.0001), while white blood cell (WBC) counts remain unchanged.
• Serum levels of cytokines (IL-6, IL-8, and IL-1) are markedly elevated in pipe smokers compared to non-smokers (p<0.0001).
• Anti-inflammatory IL-10 is significantly heightened in pipe smokers (p<0.0001), reflecting a compensatory immunomodulatory response.
• Spearman significant positive association between age and serum IL-10 levels (r=0.2242,p=0.047).
• pipe smoking leads to systemic immune dysregulation, challenging the perception of its safety relative to cigarette.

## Graphical Abstract

**Figure:**
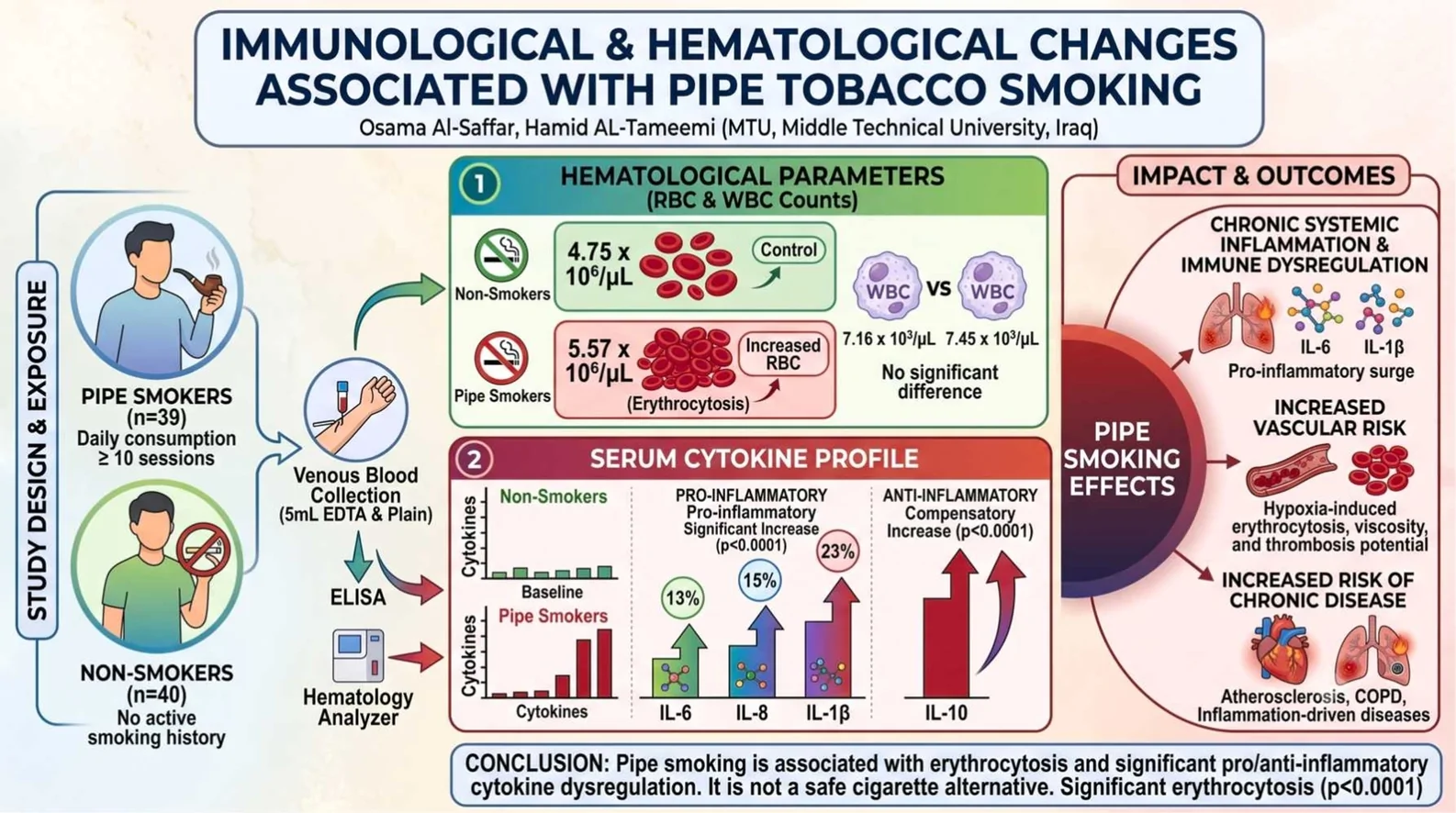

## 1 Introduction

Tobacco consumption remains a major global public health problem and a leading cause of preventable illness and death [1], [2]. Although cigarette smoke toxicity is well documented, alternative forms of tobacco use, particularly traditional pipe smoking, remain understudied. Pipe smoking involves combusting loose, cured tobacco in a bowl and drawing the smoke through a pipe stem. Therefore, findings from cigarette or waterpipe exposure cannot be directly extrapolated to pipe smokers without empirical evidence.

Traditional pipe smoking is often perceived as less harmful than cigarette smoking because pipe smokers typically hold smoke in the oral cavity without deep inhalation. However, mainstream and sidestream pipe tobacco smoke contain hazardous combustion toxicants, including volatile organic compounds, polycyclic aromatic hydrocarbons, carbon monoxide, and tobacco-specific nitrosamines, produced at varying bowl combustion temperatures [3]; National Academies of Sciences, Engineering, and Medicine [4]). Although epidemiological evidence links exclusive pipe smoking to increased risks of cardiovascular disease, respiratory disease, and aerodigestive malignancies [3], clinical evidence on systemic hematopoietic responses and circulating inflammatory cytokines in exclusive pipe smokers remains limited.

Tobacco toxicity is mediated partly through altered immune responses. Combustion smoke contains reactive oxygen species and xenobiotics that impair innate and adaptive immunity, alter immune cell activity, disrupt cytokine balance, and promote chronic inflammation. Persistent inflammatory signaling may impair pathogen clearance, delay wound healing, and reduce post-vaccination responses [5].

Studies of cigarette exposure have demonstrated altered macrophage, neutrophil, dendritic cell, and lymphocyte function, reduced phagocytic activity, and impaired antigen presentation [6]. However, comparable immunological profiles in loose-leaf pipe smokers remain largely uncharacterized.

Circulating cytokines regulate systemic inflammation. Interleukin (IL)-6, IL-8, and IL-1β are key pro-inflammatory cytokines frequently elevated in smokers and associated with chronic low-grade inflammation and increased cardiovascular and pulmonary disease risk [7]. Conversely, IL-10 is an anti-inflammatory mediator that limits excessive inflammation, and altered levels may indicate compensatory responses or immune dysregulation [8]. Cytokine profiles have been proposed as biomarkers of tobacco-induced immune disturbance, but available evidence primarily concerns cigarette or waterpipe use, leaving conventional pipe smoking understudied [9].

Tobacco smoke exposure is also associated with altered hematological indices. Cigarette smoking is associated with elevated white blood cell (WBC) counts, reflecting systemic inflammation, and increased red blood cell (RBC) counts and hematocrit, potentially because carbon monoxide-induced hypoxia stimulates erythropoiesis. These changes increase blood viscosity and may contribute to vascular strain and thrombotic risk. Although well documented in cigarette smokers, these hematological effects remain poorly characterized in exclusive pipe smokers despite exposure to combustion by-products [10], [11].

To address these gaps, we hypothesized that exclusive pipe smoking induces distinct inflammatory and hematological changes. To test this hypothesis, this study evaluated hematological and immunological profiles associated with exclusive pipe smoking. Serum IL-6, IL-8, IL-10, and IL-1β concentrations and RBC and WBC counts were compared between pipe tobacco smokers and non-smokers to identify inflammatory and hematopoietic alterations associated with this smoking practice.

## 2 Materials and methods

### 2.1 Study cohort and exposure assessment

The study included 79 adult participants: 39 exclusive pipe smokers and 40 healthy non-smokers. Group allocation was based on in-person interviews assessing detailed smoking history. Smokers were defined as daily users of traditional loose-leaf pipe tobacco for ≥ 1 continuous year who consumed ≥ 10 pipe bowls daily (mean duration: 8.45 ± 4.12 years; mean frequency: 12.30 ± 3.80 sessions/day). Smoking topography involved shallow oral puffing rather than deep inhalation. To minimize exposure misclassification, individuals who used cigarettes, shisha, e-cigarettes, heated tobacco products, or other tobacco products were excluded. Non-smokers had no lifetime smoking history or regular household or workplace exposure to secondhand smoke.

### 2.2 Inclusion and exclusion criteria

Inclusion criteria were age 18–65 years, written informed consent, and attendance at the medical center for routine occupational or periodic general health evaluations. Exclusion criteria for both groups were (1) current or previous chronic inflammatory or autoimmune disease, including rheumatoid arthritis, systemic lupus erythematosus, or inflammatory bowel disease; (2) active or recent acute infection within 4 weeks; (3) history of malignancy, hematological abnormalities, or cardiovascular disease; (4) major surgery, trauma, or blood donation within 3 months; (5) corticosteroid, non-steroidal anti-inflammatory drug (NSAID), or immunomodulatory therapy within 30 days; (6) history of chronic alcohol abuse; and (7) pregnancy or lactation.

### 2.3 Participant recruitment and sampling design

From October 2024 to July 2025, participants were consecutively enrolled at Al-Andalus Oncology Hospital through convenience sampling during routine occupational health checks and periodic outpatient medical evaluations. Both groups were recruited from the same institutional setting to maintain comparable demographic characteristics and reduce environmental selection bias. Because exclusive conventional pipe smoking is relatively uncommon, the sample size was determined pragmatically based on strict eligibility criteria and the 10-month recruitment period. A post hoc power calculation based on observed differences in primary cytokine concentrations indicated > 90% statistical power (β = 0.10, α = 0.05) to detect the observed between-group differences.

### 2.4 Specimen collection

A total of 5 mL of venous blood was collected from each participant using sterile Vacutainer tubes. For complete blood count analysis, 2 mL was transferred into EDTA tubes and analyzed using an automated hematology analyzer (Sysmex XN-1000, Sysmex Corporation, Kobe, Japan). The remaining 3 mL was collected in plain tubes, allowed to clot for 30 min at room temperature, and centrifuged at 3000 rpm for 10 min. Serum was separated and stored at −80 °C until cytokine analysis.

### 2.5 Measurement of cytokines

Serum IL-6, IL-8, IL-10, and IL-1β concentrations were quantified using commercial quantitative sandwich enzyme-linked immunosorbent assay (ELISA) kits (Quantikine, R&D Systems, Minneapolis, MN, USA; Catalog Nos. D6050 for IL-6, D8000C for IL-8, D1000B for IL-10, and DLB50 for IL-1β) according to the manufacturer's instructions. Minimum detectable concentrations were 0.70 pg/mL for IL-6, 3.50 pg/mL for IL-8, 3.90 pg/mL for IL-10, and 1.00 pg/mL for IL-1β. Specimens and recombinant human standards were assayed in duplicate. Absorbance was measured at 450 nm with wavelength correction at 570 nm using a BioTek ELx800 microplate reader (BioTek Instruments, Winooski, VT, USA). Cytokine concentrations were interpolated from four-parameter logistic (4-PL) standard curves. Intra-assay and inter-assay coefficients of variation (CV%) were < 6.5% and < 8.5%, respectively.

### 2.6 Statistical analysis

Statistical analyses were performed using GraphPad Prism version 9.0 (GraphPad Software, San Diego, CA, USA). Normality of continuous variables was assessed using the Shapiro–Wilk test, and homogeneity of variance using the F-test. Normally distributed variables are presented as mean ± standard deviation (SD). Differences between pipe smokers and non-smokers were assessed using two-tailed unpaired Student's *t*-tests, with Welch's correction for unequal variances. Categorical demographic variables (biological sex and ethnicity) were compared using Fisher's exact test or chi-square analysis. Associations between age and circulating cytokine concentrations were assessed using two-tailed Spearman rank correlations (r). Between-group mean differences for all primary endpoints were reported with 95% confidence intervals (CIs), and statistical significance was set at two-tailed p < 0.05. The primary hypothesis assessed between-group differences in cell counts and cytokine concentrations, whereas age–cytokine correlations were exploratory. Bonferroni correction for four cytokine correlations yielded an adjusted α = 0.0125; therefore, the observed age–IL-10 correlation (p = 0.047) was considered hypothesis-generating rather than definitive.

## 3 Results

Table 1 summarizes the baseline demographic and clinical characteristics of the participants.

**Table 1:** Baseline demographic, clinical, and smoking characteristics of the study cohort.

**Characteristic**	**Non-Smokers** **(n = 40)** Mean ± SD	**Pipe Smokers** **(n = 39)** Mean ± SD	***p*****-value**
**Age (years)**	38.6 ± 11.2	38.1 ± 14.3	0.862^a^
**Gender** n (%)			0.821^b^
**Male**	21 (52.5%)	21 (53.8%)	
**Female**	19 (47.5%)	18 (46.2%)	
**Ethnicity** n (%)			
**Arab / Iraqi**	40 (100%)	39 (100%)	> 0.999^b^
**BMI (kg/m**^**2**^**)**	25.42 ± 3.18	25.88 ± 3.45	0.541^a^
**Smoking History**			—
**Duration of smoking (years)**	N/A	8.45 ± 4.12	—
**Pipe sessions/day**	N/A	12.30 ± 3.80	—

Data are presented as Mean ± standard deviation (SD) or count (percentage).

^a^ Evaluated using unpaired two-tailed Student’s *t*-test.

^b^ Evaluated using Chi-square / Fisher's exact test.

BMI: Body Mass Index; N/A: Not applicable.

Sex distribution was comparable between groups, with 21 males and 18 females among pipe smokers and 21 males and 19 females among controls (*p* = 0.821; Table 1, Fig. 2). Mean age also did not differ significantly between pipe smokers and non-smokers (38.1 ± 14.3 *vs.* 38.6 ± 11.2 years; *p* = 0.862; Table 1, Fig. 1).

**Fig. 1:**
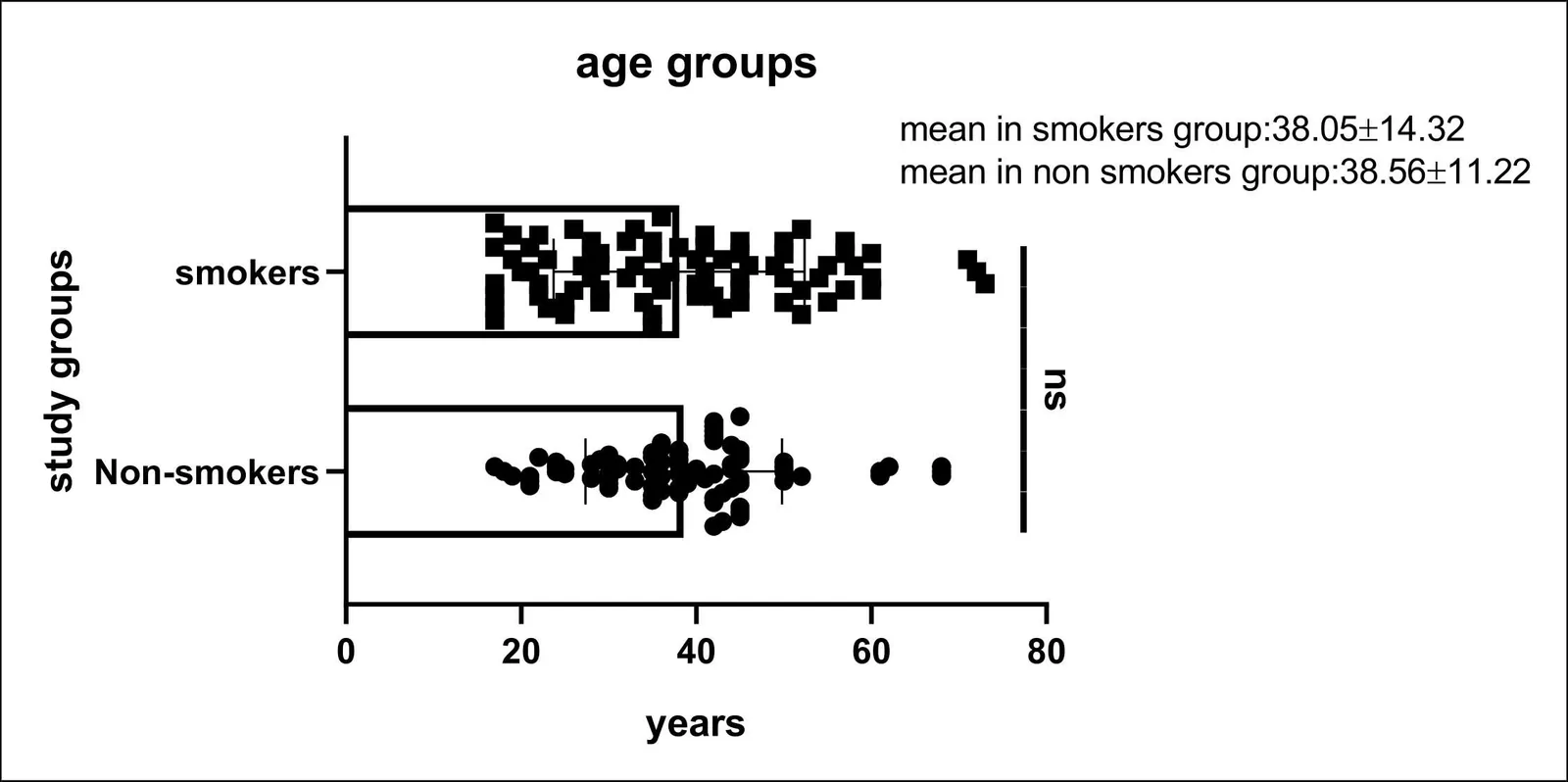
Distribution of chronological age among exclusive pipe tobacco smokers (n = 39) versus healthy non-smoking controls (n = 40). Comparisons were assessed using an unpaired two-tailed Student’s *t*-test (*p* = 0.862).

**Fig. 2:**
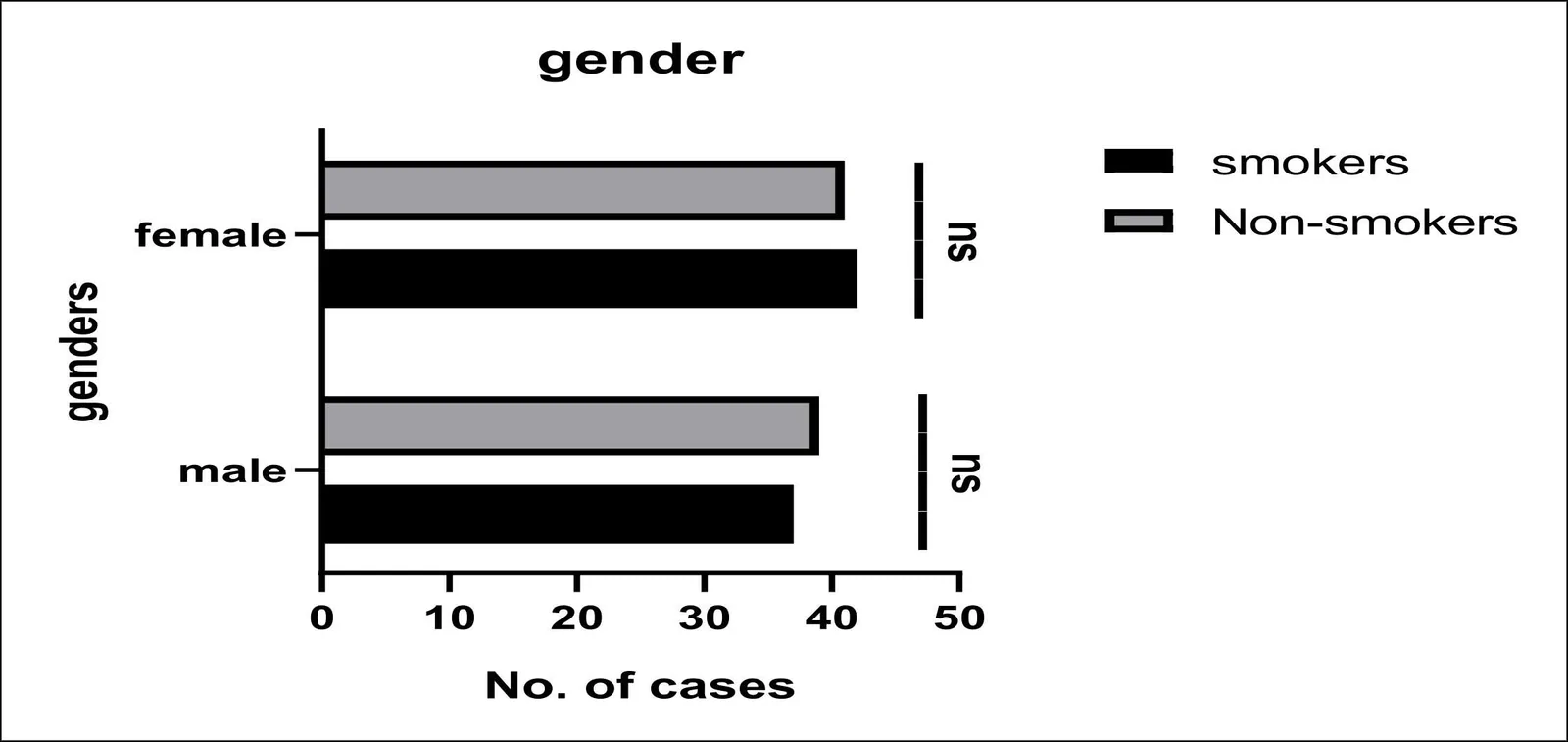
Distribution of biological sex among pipe tobacco smokers and non-smokers. Assessed using the Chi-square test (*p* = 0.821).

RBC counts were significantly higher among pipe smokers than non-smokers (5.57 ± 0.99 *vs*. 4.75 ± 0.79 × 10⁶/μL; mean difference: 0.82 × 10⁶/μL, 95% CI: 0.42–1.22; *p* < 0.0001; Fig. 3). In contrast, total WBC counts did not differ significantly between groups (7.45 ± 1.84 *vs*. 7.16 ± 1.42 × 10 ³/μL; mean difference: 0.28 × 10 ³/μL, 95% CI: −0.45–1.02; *p* = 0.443; Fig. 4).

**Fig. 3:**
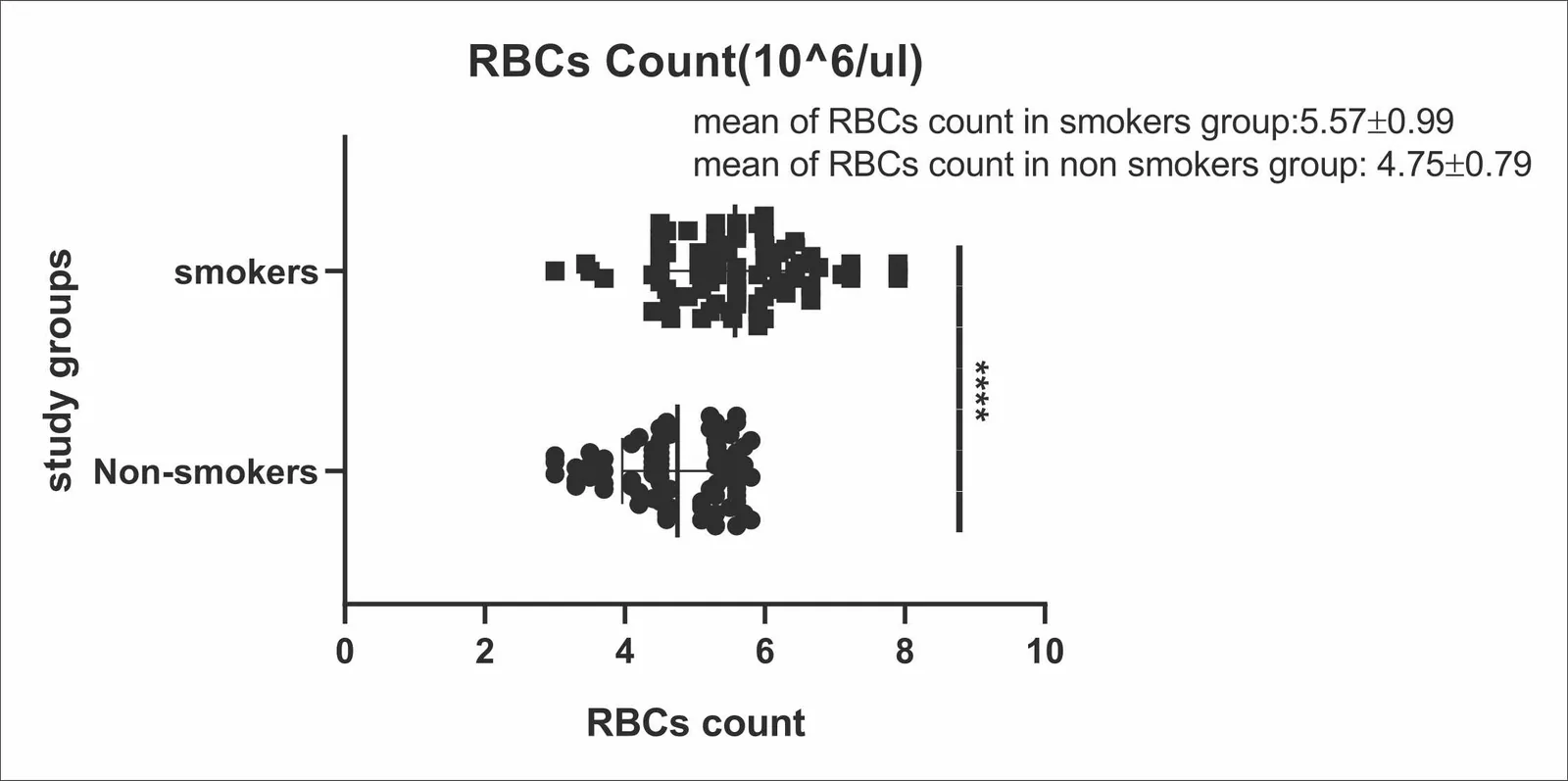
Comparative red blood cell (RBC) counts, indicating a significant elevation among pipe smokers relative to non-smokers (mean difference: +0.82 ×10⁶/μL, 95% CI: 0.42–1.22; p < 0.0001, assessed via unpaired Student’s *t*-test).

**Fig. 4:**
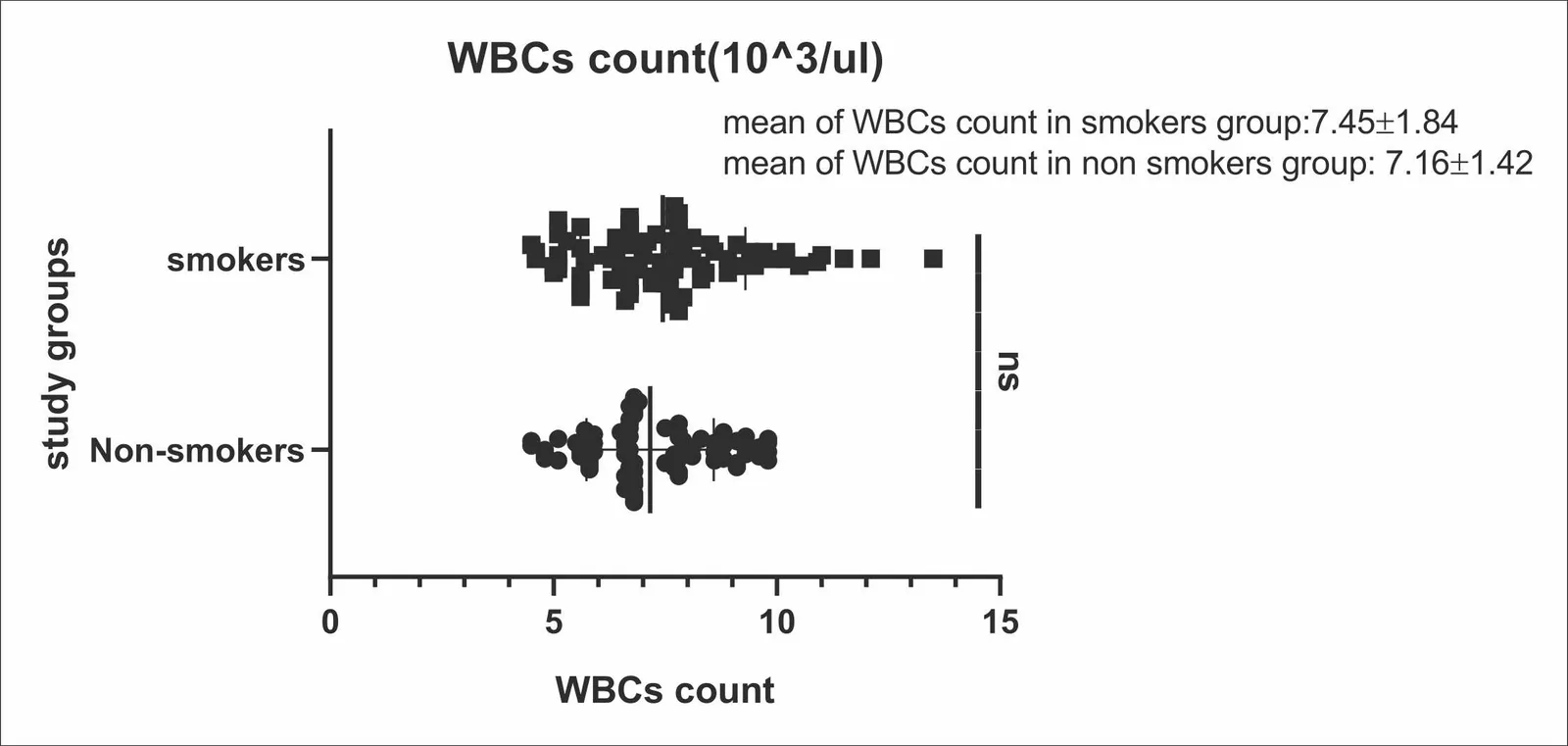
Total white blood cell (WBC) counts, indicating no statistically significant difference between the cohorts (*p* = 0.443).

All measured cytokines were significantly higher among pipe smokers than non-smokers (Fig. 5, Fig. 6, Fig. 7, Fig. 8): IL-6, (*p* < 0.0001); IL-8, (*p* < 0.0001); IL-1β, (*p* < 0.0001); and IL-10, (*p* < 0.0001).

**Fig. 5:**
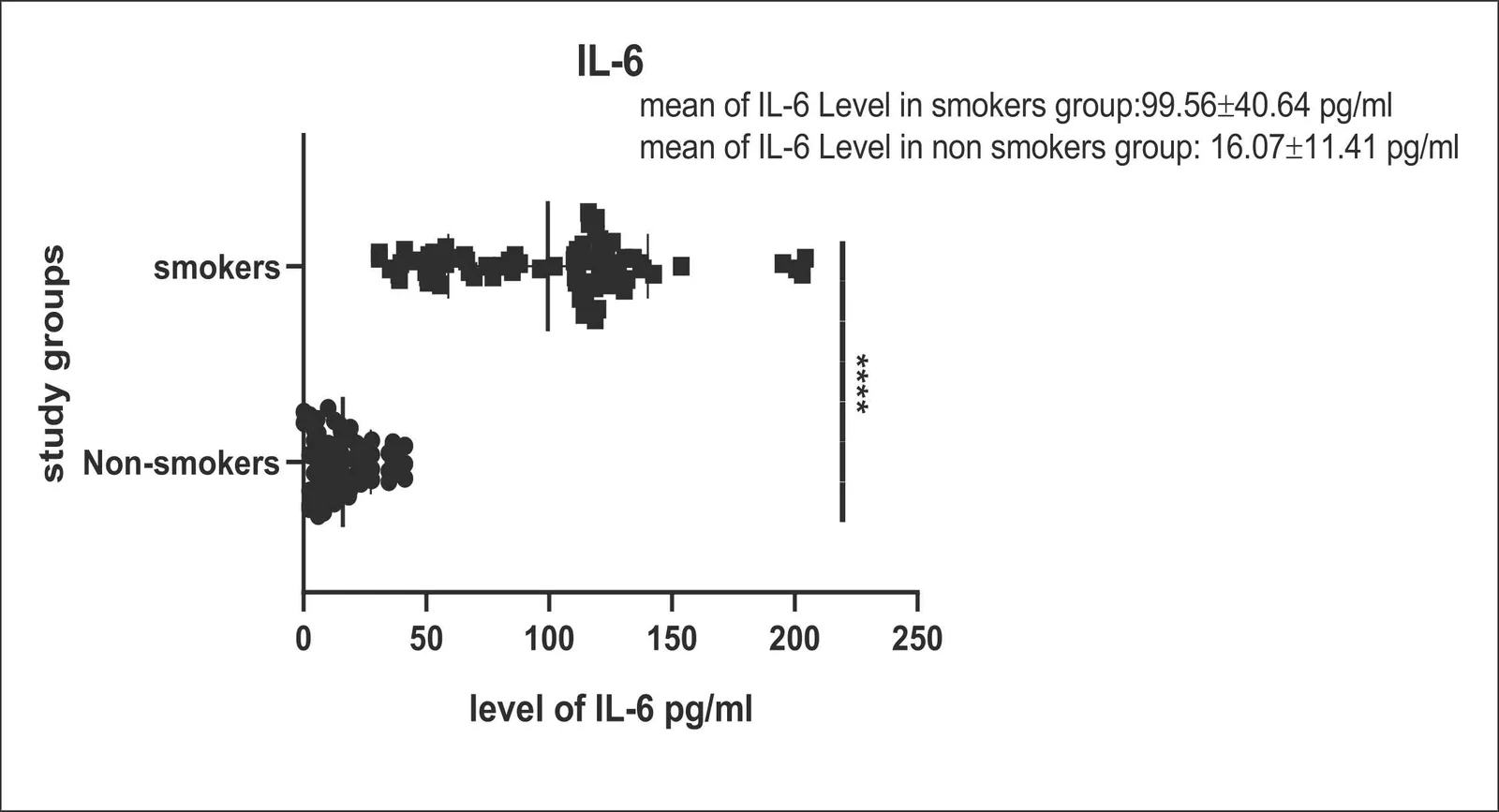
Serum interleukin-6 (IL-6) concentrations, demonstrating a marked elevation among pipe smokers relative to non-smokers (mean difference: +83.49 pg/mL, 95% CI: 70.07–96.91; *p* < 0.0001).

**Fig. 6:**
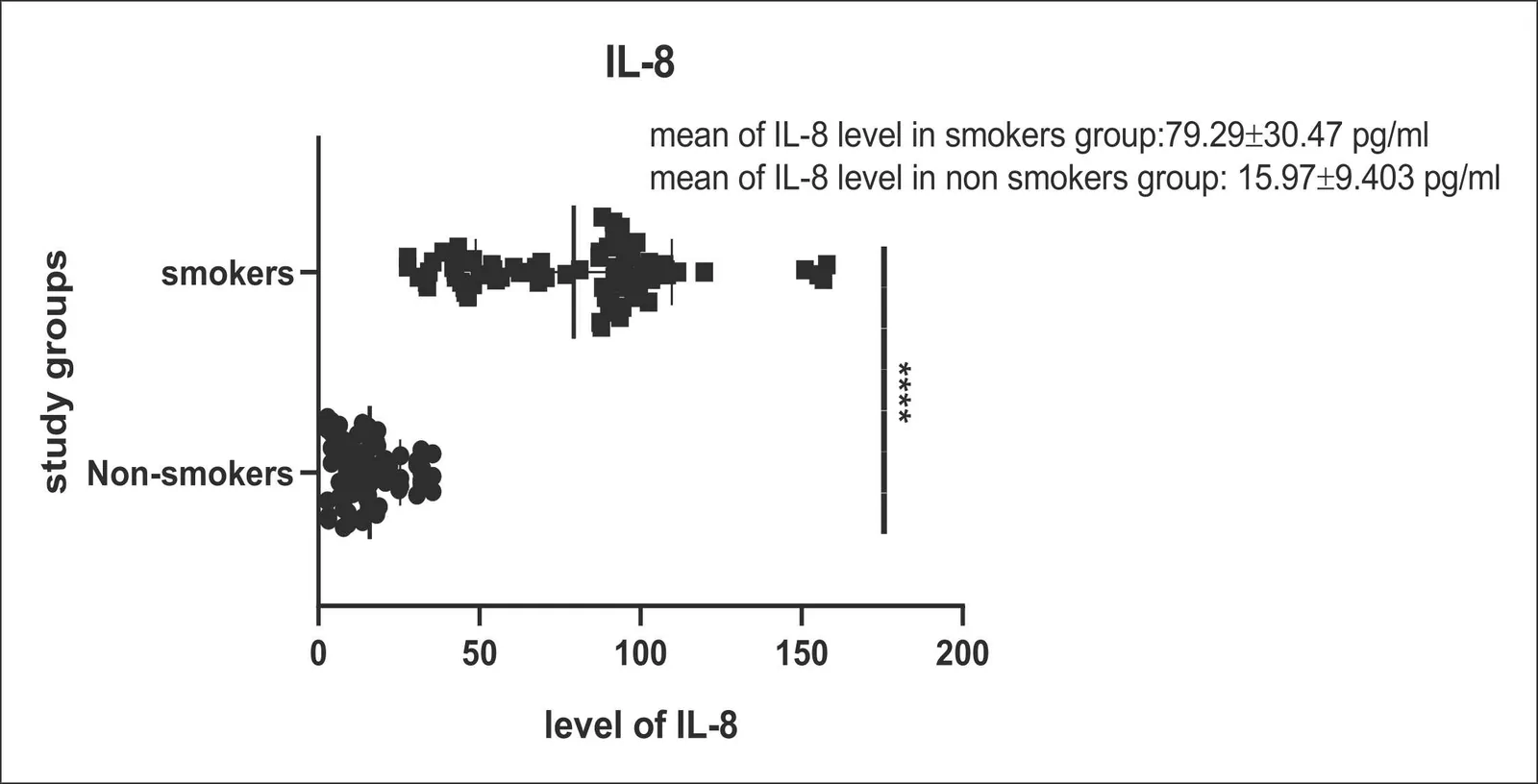
Serum interleukin-8 (IL-8) concentrations, demonstrating a marked elevation among pipe smokers relative to non-smokers (mean difference: +63.32 pg/mL, 95% CI: 53.15–73.49; *p* < 0.0001).

**Fig. 7:**
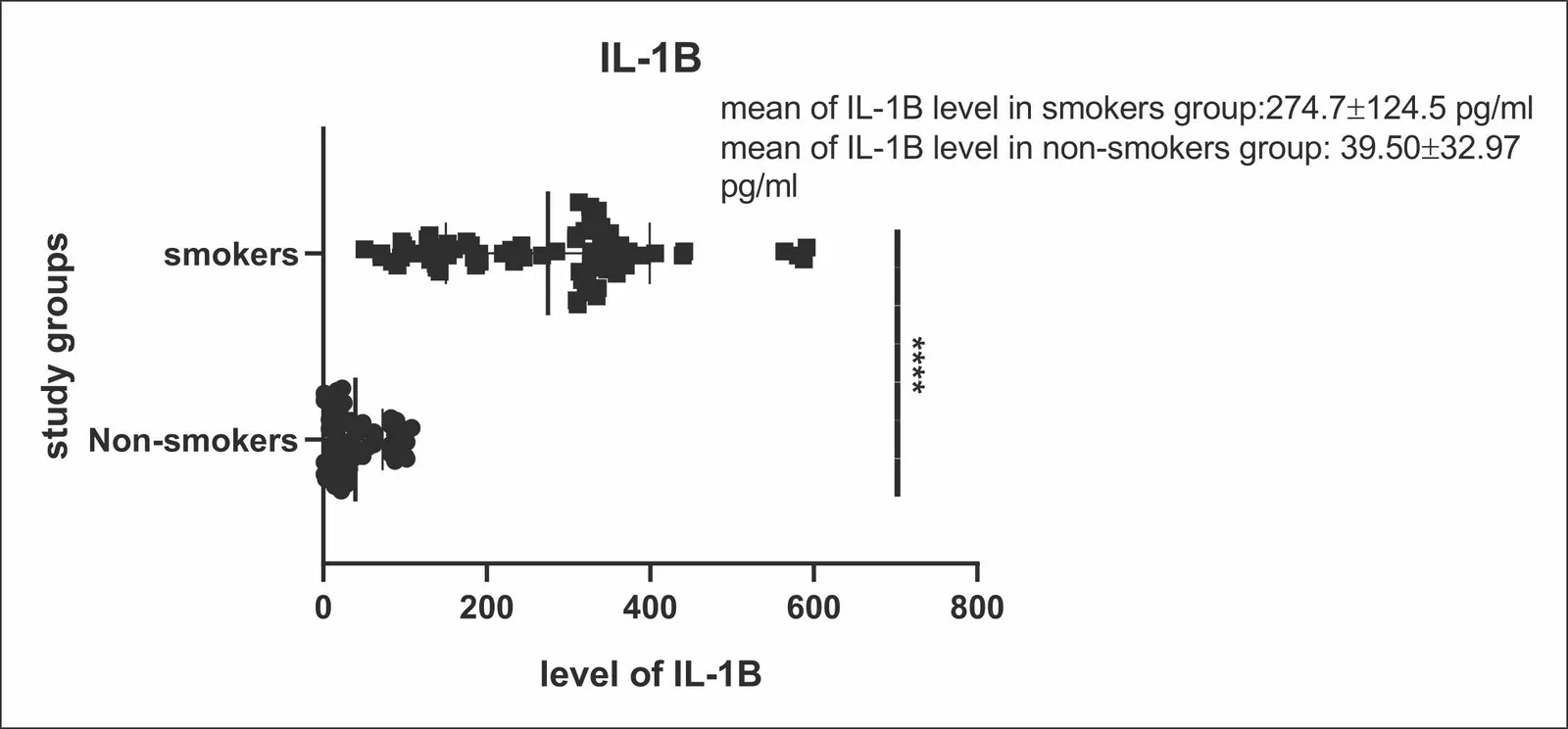
Serum interleukin-1β (IL-1β) concentrations, demonstrating a marked elevation among pipe smokers relative to non-smokers (mean difference: +235.2 pg/mL, 95% CI: 194.2–276.2; *p* < 0.0001).

**Fig. 8:**
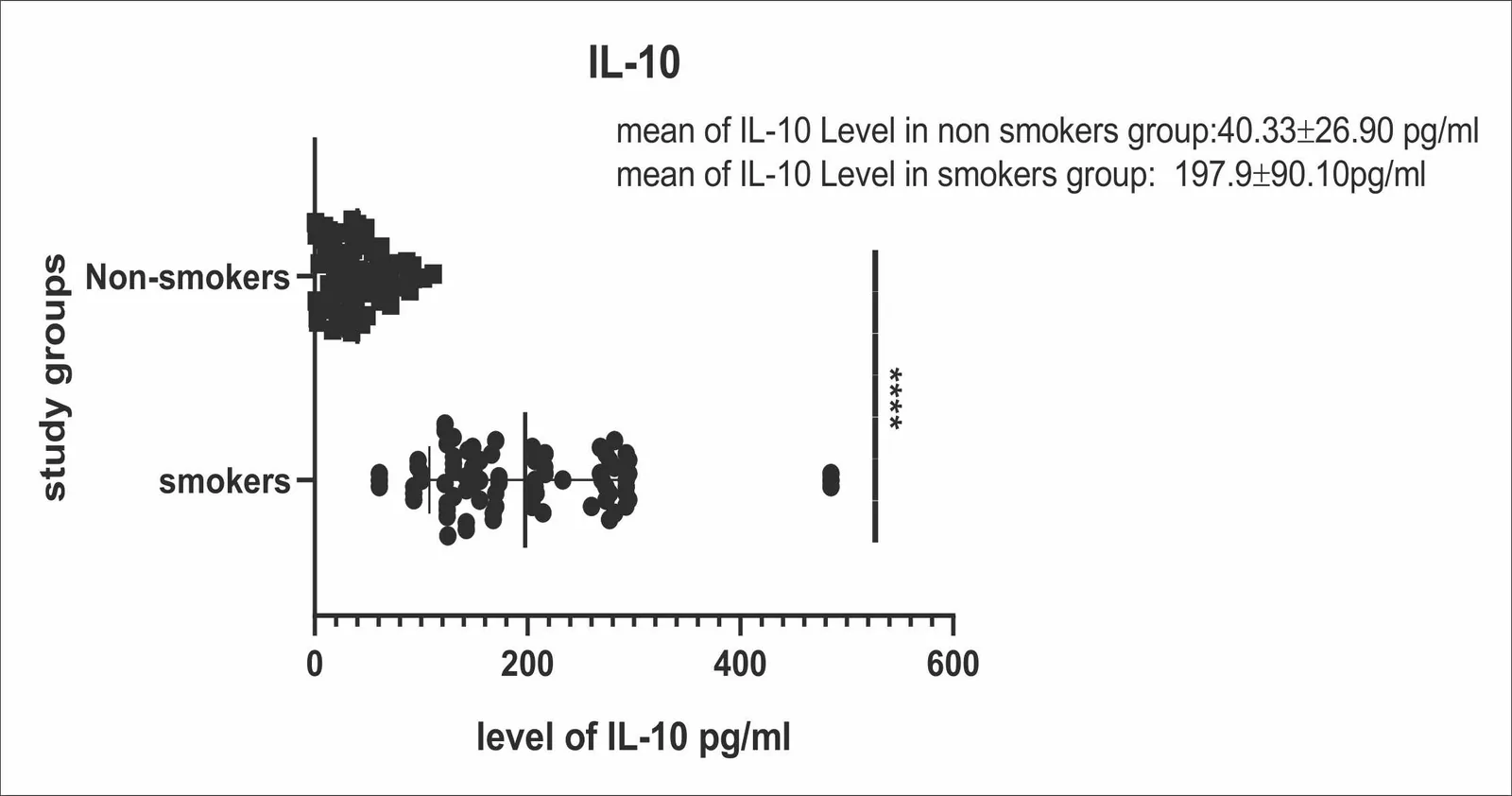
Serum interleukin-10 (IL-10) concentrations, demonstrating a marked elevation among pipe smokers relative to non-smokers (mean difference: +157.6 pg/mL, 95% CI: 127.7–187.5; *p* < 0.0001).

Spearman correlation analysis assessed associations between age and serum IL-6, IL-8, IL-10, and IL-1β levels (Table 2). Weak, non-significant positive correlations were observed for IL-6 (r = 0.1044, p = 0.360), IL-8 (r = 0.1092, p = 0.338), and IL-1β (r = 0.03944, p = 0.730). Age was weakly correlated with IL-10 (r = 0.2242, p = 0.047, r² = 0.050), indicating that age explained approximately 5% of the variance in IL-10 levels. However, this association did not meet the Bonferroni-adjusted significance threshold (α = 0.0125) and was therefore considered exploratory.

**Table 2:** Correlation between age and serum cytokine levels (IL-6, IL-8, IL-10, and IL-1β).

Parameter	Age *vs*. IL-6	Age *vs*. IL-8	Age *vs*. IL-10	Age *vs*. IL-1Β
Spearman r	0.1044	0.1092	0.2242	0.03944
95% confidence interval	–0.1260–0.3242	–0.1213–0.3284	–0.003446–0.4297	–0.1897–0.2645
*p*-value (two-tailed)	0.360	0.338	0.047	0.730
*p*-value summary	ns	ns	*	ns
Exact or approximate *p*-value	Approximate	Approximate	Approximate	Approximate
Significant (α = 0.05)	No	No	Yes	No
Number of XY pairs	79	79	79	79

## 4 Discussion

This study compared RBC and WBC counts and serum IL-6, IL-8, IL-1β, and IL-10 levels between pipe smokers and non-smokers. Age and biological sex distributions did not differ significantly between groups (Table 1), reducing major baseline demographic differences. However, because multivariable models were not applied, residual confounding by unmeasured lifestyle factors cannot be excluded.

Pipe smokers had significantly higher circulating erythrocyte counts than controls. Toxicological models indicate that inhalation of combustion emissions, particularly carbon monoxide, promotes carboxyhemoglobin formation, causing functional hypoxia and compensatory erythropoiesis [12], [13]. Although reactive erythrocytosis may increase blood viscosity and vascular resistance, this study did not measure carboxyhemoglobin, circulating erythropoietin, or rheological parameters to confirm this mechanism. Nevertheless, the observed increase provides preliminary clinical evidence of altered erythropoiesis associated with traditional pipe smoking.

Total WBC counts did not differ significantly between pipe smokers and controls. Although tobacco inhalation commonly induces peripheral leukocytosis [14], [15], an unchanged total WBC count does not exclude immune alterations because it may obscure changes in specific leukocyte populations. Differential leukocyte counts were not measured, and the modest sample size may also have limited the ability to detect subtle differences.

Serum IL-6 and IL-8 levels were markedly higher among pipe smokers. IL-6 is a key pro-inflammatory mediator of hepatic acute-phase responses [16], [17], whereas IL-8 promotes neutrophil chemotaxis to sites of inflammation [18]. Their concurrent elevation suggests systemic inflammatory activation associated with pipe smoking, with potential implications for chronic vascular and tissue pathology.

Circulating IL-1β concentrations were also markedly higher in pipe smokers. As an early-response cytokine, IL-1β regulates systemic inflammation, fever, leukocyte recruitment, and tissue remodeling [19]. This elevation indicates sustained pro-inflammatory signaling in habitual pipe users, potentially increasing the risk of atherosclerosis, chronic obstructive pulmonary disease (COPD), and related immune-mediated conditions [20], [21]. Persistent IL-1β release may reflect activation of pattern-recognition pathways, including inflammasome assembly, by toxic pipe smoke pyrolysis products, promoting local and systemic cellular stress.

Serum IL-10 titers were also significantly higher in pipe smokers than in non-smokers. IL-10 is a protective counter-regulator that limits tissue injury by suppressing antigen presentation and macrophage effector cytokines [22]). This elevation may compensate for increased IL-6, IL-8, and IL-1β; however, concurrent increases in opposing cytokine subsets suggest dysregulated immune activation rather than effective anti-inflammatory control. Simultaneous activation of effector and regulatory pathways indicates persistent immune activation and insufficient regulation during chronic exposure to combustion toxicants.

Spearman rank testing showed a weak positive correlation between participant age and circulating IL-10 concentration (*r* = 0.2242, *p* = 0.047). Despite marginal significance, the low coefficient of determination (*r* = 0.05) indicates that age explains little variation in IL-10, warranting cautious interpretation. This weak association may reflect immunosenescent changes, whereas the marked IL-10 elevation in pipe users likely reflects chronic tobacco combustion exposure rather than chronologic age. The absence of correlations between age and pro-inflammatory markers (IL-6, IL-8, and IL-1β) further suggests that pipe-related inflammatory activation is independent of participant age.

Collectively, these findings show that traditional pipe smoking alters hematological and cytokine profiles. Elevated erythrocyte counts may increase long-term vascular risk, while concurrent increases in effector and regulatory cytokines indicate sustained immune stress. This dysregulation is consistent with mechanisms underlying tobacco-associated vascular disease, chronic obstructive airway changes, and oncogenesis. Simultaneous elevation of inflammatory and counter-regulatory mediators suggests systemic immune disruption rather than isolated pro-inflammatory activation, potentially explaining smokers’ concurrent susceptibility to opportunistic infections and inflammation-driven organ damage.

## 5 Study limitations

Although this study provides comparative data on exclusive pipe users, several limitations should be considered. The cross-sectional design captures biological parameters at a single time point, precluding causal inference between smoke exposure and immune alterations. The small, single-center cohort (n = 79) limited subgroup analyses and may restrict generalizability. Exposure classification relied on self-reported histories without biochemical verification, such as cotinine, exhaled carbon monoxide, or carboxyhemoglobin measurements, or standardized cumulative indices such as pipe-years. Hematological profiling was limited to basic cell counts and excluded leukocyte function, hematocrit, and rheological parameters that could clarify underlying mechanisms. Unmeasured lifestyle factors, including diet and occupational exposures, may also have confounded the findings. Longitudinal studies integrating objective exposure biomarkers with multiparameter cellular phenotyping are needed to characterize temporal relationships. In addition, the inflammatory variation of the menstrual cycle in females was not evaluated, corresponding to an unmeasured confounding factor.

## 6 Conclusion

This cross-sectional study showed that habitual pipe smoking was associated with elevated erythrocyte counts and increased pro-inflammatory (IL-6, IL-8, and IL-1β) and regulatory (IL-10) cytokines. These changes indicate systemic immune activation and sustained hematopoietic stimulation, suggesting that pipe smoking is not biologically benign. Although prospective studies are needed to assess long-term clinical outcomes, these findings question the presumed relative safety of pipe smoking and support rigorous regulatory surveillance comparable to that for other forms of combusted tobacco.

## CRediT authorship contribution statement

**Osama Al-Saffar:** Writing – original draft, Supervision, Methodology, Investigation, Data curation, Conceptualization. **Hamid AL-Tameemi:** Software, Formal analysis, Data curation.

## Funding statement

This research received no specific grant from any funding agency in the public, commercial or not-for-profit sectors.

## Conflict of interest

The authors declare no conflict of interest.

## Declaration of Competing interest

The authors declare that they have no known competing financial interests or personal relationships that could have appeared to influence the work reported in this paper.

## Data Availability

Data will be made available on request.
